# Agreement between cranial and facial classification through clinical observation and anthropometric measurement among envigado school children

**DOI:** 10.1186/1472-6831-14-50

**Published:** 2014-05-07

**Authors:** Adriana M Torres-Restrepo, Ana M Quintero-Monsalve, Juan F Giraldo-Mira, Zulma Vanessa Rueda, Natalia Vélez-Trujillo, Paola Botero-Mariaca

**Affiliations:** 1 Universidad Cooperativa de Colombia, Carrera 47 # 37 sur 18. Envigado, Antioquia, Medellín, Colombia

**Keywords:** Anthropometry, Craniofacial type, Face anatomy, Head anatomy

## Abstract

**Background:**

To evaluate the agreement between cranial and facial classification obtained by clinical observation and anthropometric measurements among school children from the municipality of Envigado, Colombia.

**Methods:**

This cross-sectional study was carried out among 8-15-year-old children. Initially, an indirect clinical observation was made to determine the skull pattern (dolichocephalic, mesocephalic or brachycephalic), based on visual equivalence of right eurion- left eurion and glabella-opisthocranion anthropometric points, as well as the facial type (leptoprosopic, mesoprosopic and euryprosopic), according to the left and right zygomatic, nasion and gnation points. Following, a direct measurement was conducted with an anthropometer using the same landmarks for cranial width and length, as well as for facial width and height. Subsequently, both the facial index [euryprosopic (≤80.9%), mesoprosopic (between 81% - 93%) and leptoprosopic (≥93.1%)] and the cranial index [dolichocephalic (index ≤ 75.9%), mesocephalic (between 76% - 81%), and brachycephalic (≥81.1%)] were determined. Concordance between the indices obtained was calculated by direct and indirect measurement using the Kappa statistic.

**Results:**

A total of 313 students were enrolled; 172 (55%) were female and 141 (45%) male. The agreement between the direct and indirect facial index measurements was 0.189 (95% CI 0.117-0261), and the cranial index was 0.388 (95% CI 0.304-0.473), indicating poor concordance.

**Conclusions:**

No agreement was observed between direct measurements conducted with an anthropometer and indirect measurements via visual evaluation. Therefore, the indirect visual classification method is not appropriate to calculate the cranial and facial indices.

## Background

Post-natal craniofacial growth and development is characterized by an increase in the width and length of both the face and the skull, as well as by a significant change in the proportions of these, resulting in morphological variations in the three planes of space (vertical, transverse and antero-posterior), until skeletal maturity is reached [1]. To assess both the head and the face, measurements can be conducted that yield cranial and facial classification, using indices associated with growth patterns, which make orthopedic and/or orthodontic diagnosis and treatment planning easier.

In the early twentieth century, the first orthodontists began to quantitatively determine structural changes of facial skeleton through X-rays. In 1931, Holly Broadbent introduced basic techniques in living subjects’ cephalometric evaluation, recording images of both hard and soft- tissues. Cephalometry, then, becomes an indirect form of facial anthropometry [2,3]. Apart from the latter, indirect measurements include: visual clinical assessment, craniofacial photography, and 3D scanning [4].

Anthropometry of soft- tissue using a measurement instrument (anthropometer) is considered a direct quantitative method. Advantages of this technique include its non-invasive nature and its allowance to access to areas covered by hair (e.g., head circumference, width, length and height) or to areas that, otherwise, would be observed distorted through indirect anthropometry (e.g., face depth in photography) [4].

Early in his career as a surgeon, Leslie Farkas was dissatisfied with the determination of the morphologic changes in the head and face by visual assessment. Therefore, he began to explore the use of classic anthropometric methods for quantitative analysis of faces, pre and postoperatively, and thus establish the differences between direct and indirect measurement methods with clinical assessment [5,6].

Some studies have showed a continuous change of the facial and cranial indices with growth [7], essentially in males (Deutsch population), but others report that between 10–20 years of age, little change can be found (American population). Growth of upper craniofacial region shows a rapid development phase in the first year of life, significant growth up to the fifth year, and it is virtually complete at age 6 [8,9]. Facial growth achieves 40% at birth and 65% at age 7; from there to 10 years, the change is 15% in bizygomatic width, that has 80% of its full growth at age 7 [10].

Many clinicians, in the course of their practice, conduct craniofacial complex classification subjectively by visual assessment. However, performing direct measurement not only allows them to confirm the diagnosis, but also reliably provides facial and cranial indices. The aim of this study was to evaluate the agreement between cranial and facial classification obtained by clinical observation and anthropometric measurement in school children between 8- 15-year- old from the municipality of Envigado, Colombia.

## Methods

### Type of study: cross-sectional

#### Population

Boys and girls enrolled in public and urban educational institutions in the municipality of Envigado, Colombia. Inclusion criteria: 1) school-aged children between 8–15 years old who had no craniofacial asymmetries; 2) school-aged children who had not had, or that at the time of the evaluation, did not present active orthodontic/orthopedic treatment; and 3) authorization of the school-aged child’s father/mother or guardian to be part of the study, as well as their signed consent form and assent. Exclusion criteria: 1) school-aged children with syndromes or traumas affecting the craniofacial complex; or 2) school-aged children with neurological and psychiatric disorders, since these might affect understanding and signing of the written consent form. This study is in compliance with the ethical requirements provided by the Resolution 8430 of 1993, issued by the Ministry of Health of Colombia, and was approved by the Ethics Committee of the Universidad Cooperativa de Colombia.

#### Sample collection

Prior to the beginning of the study, an intra and interobserver standardization was carried out among the three researchers who were to assess children with the researcher leader, in order to identify anthropometric points, visual assessment and measurement using an anthropometer. In addition, a pilot test was conducted to calibrate the measuring instrument, the data collection form, as well as the whole assessment process in order to make corrections where required. Amongst 21 existing urban institutions in the municipality of Envigado, five were randomly selected. Upon authorization from the Secretariat of Education of Envigado and in accordance with the requirements put forth by each educational institution, a complete list of the different groups of students by grade was made to choose eligible children for the study. Only one entity allowed holding a meeting with parents and children selected, in order to explain what the project consisted about and to answer questions concerning the process. Subsequently, in the presence of the researchers and two witnesses, the minor’s parent or guardian signed a consent form and assent. For the other institutions, an explanatory and a consent forms and assent were distributed to be completed and signed by the parents and two witnesses.

#### Measurement processes

In order to avoid interobserver errors in measurements, one researcher conducted the indirect visual evaluation, while a different researcher carried out the direct assessment, using an anthropometer. Initially, the child was seated on a chair, with the Camper plane parallel to the ground and the vertical mid-facial axis perpendicular to the ground, with the mandible in the maximum intercuspal position and with the mouth closed. The examiner had a fix distance of 20-cm and had eye-to-eye level with the patient.

**Figure 1:**
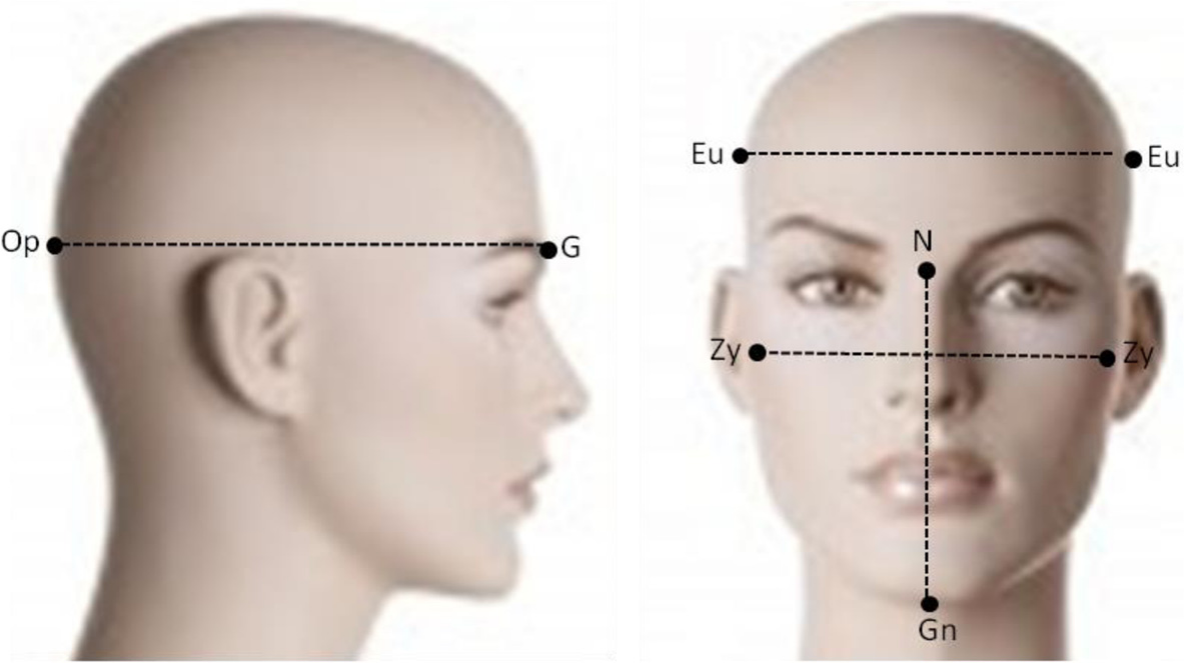
**Facial height: It is the distance in mm between N = nasion (point of intersection of the frontonasal and intranasal sutures) and Gn = gnation (the most anterior-inferior point of the chin contour).** Face width: It is the distance in mm between left zygomatic (the most prominent point of the zygomatic bone) and right zygomatic = Zy. Cranial length (antero-posterior length): It is the distance in mm between G = glabella (the most prominent point of the frontal bone in the midsagittal plane between the brow ridges) and Op = Opisthocranion (point of the occipital squama, which in the sagittal plane, is located furthest from the glabella point). Cranial width: It is the distance in mm between left Eurion (the most lateral point of the neurocranium) and right Eurion = Eu.

For the indirect visual measurement, the researcher stood a step away in front of the child and conducted the profile measurement. Then, he marked the skull type observed in the data collection form with an X cross (dolichocephalic, mesocephalic or brachycephalic), based on visual equivalence of right eurion-left eurion and glabella-opisthocranion anthropometric landmarks; and the facial type (leptoprosopic, mesoprosopic and euryprosopic), according to the left and right zygomatic, nasion and gnation points (Figure 1).

For direct measurement, the other researcher used the same landmarks and measured them using an anthropometer, pressing the tip against the surface of the underlying bone on bone landmark, and recorded the measurement in millimeters on the data collection form. The facial index was determined by dividing the face height (from nasion to gnation) by its width (from right zygomatic to left zygomatic) and the result was multiplied by 100; then, it was classified as follows [3]: euryprosopic (≤80.9%), mesoprosopic (between 81% - 93%), and leptoprosopic (≥93.1%). For the cranial index, the relationship between the head’s maximum width or transverse diameter (from right eurion to left eurion) and the head’s maximum length or antero-posterior diameter (from opisthocranion and glabella) was determined. The maximum width was divided by the maximum length and the result was multiplied by 100 to express it as a percentage. The resulting data were classified in reference to the index developed by Anders Retzius [11,12]: dolichocephalic (index ≤ 75.9%), mesocephalic (between 76% - 81%), and brachycephalic (≥81.1%).

#### Sample size

Multiple sample sizes were calculated for agreement studies, both for cranial and facial indices. Those with the greatest number of children required were used. For the cranial index, the highest number was obtained with an expected agreement of 0.25 and a random agreement proportion of 0.546 (n = 74). For the facial index, an agreement of 0.25 and a ratio of random agreement proportion of 0.488 (n = 59) was obtained. With an increase of sample size of 10%, due to missing data, the number of children to be assesses were 146.

#### Statistical analyses

Five months after completion of the sample collection, and in order to evaluate intraobserver agreement, a second measurement process was performed both qualitatively and quantitatively using an anthropometer. For this purpose, a researcher in charge of the entire methodological part different from those who conducted direct and indirect measurements, randomly selected 12.5% (40/313) of the infants already assessed, and made a list with the names of those children who were to be remeasured, following the procedures described above. The researcher who made the measurement filled out the information in data collection form and sent it to the methodological investigator. People who made measurements never had contact with information processing, and simply interpreted the results processed by the person responsible for methodology and study analysis. To assess intraobserver concordance between facial and cranial indices reported by indirect assessment, Cohen’s Kappa coefficients were estimated. To calculate intraobserver agreement between the facial and cranial indices measured using an anthropometer, the intraclass correlation coefficients were estimated using a two-factor mixed effects model.

Subsequently, prior to analysis, a quality control of the database was performed. For this purpose, 10% of all children included in the study were taken and the information in the data collection form was cross-checked with the data typed in Excel®. The data analysis was performed using SPSS® Statistics v20.0 (SPSS Inc., Chicago, Il, USA) software. The results of the cranial and facial indices, both through visual evaluation and using an anthropometer, are reported as absolute and relative frequencies with their corresponding percentages. The agreement between facial and cranial indices, obtained by direct and indirect assessment, were estimated by calculating the percentage of agreement and Cohen’s Kappa coefficient. The agreement was interpreted as poor when the calculated values ranged between 0.0- 0.40, moderate between 0.41 - 0.6, good between 0.61- 0.8, and almost perfect > 0.8. Negative values were interpreted as equal to 0.0.

## Results

A total of 750 students were given a consent form and assent. Within these, 273 did not take the consent form home, 148 parents did not authorize participation in the study, and 16 were under active orthodontic treatment. Finally, 313 students were admitted to the study: 172 (55%) female and 141 (45%) male. The age distribution was as follows: 8 years (n = 20), 9 years (n = 45), 10 years (n = 51), 11 years (n = 52), 12 years (n = 47), 13 years (n = 24), 14 years (n = 42), and 15 years (n = 32). 42.2% of the population belonged to socioeconomic stratum two, 31.3% to stratum three, 9.9% to stratum one, 3.8% to stratum four and 40 students did not report this information.

Table 1 shows the frequency of students classified according to the facial index, both qualitatively and quantitatively. In both types of measurements, direct and indirect, the mesoprosopic type was the most prevalent (47.9% and 40.3%, respectively). However, in the indirect measurement, the most dominant type was the euryprosopic (26.8%), compared with the direct measurement (4.2%).

**Table 1:** Frequency of facial categories obtained by qualitative and quantitative measurement in school children between 8–15 years from Envigado, Colombia

	**Direct measurement (quantitative)**		**Indirect measurement (qualitative)**	
	**Frequency**	**Percentage**	**Frequency**	**Percentage**
Euryprosopic	13	4.2	84	26.8
Mesoprosopic	150	47.9	126	40.3
Leptoprosopic	150	47.9	103	32.9
Total	313	100	313	100

Table 2 shows the frequency of students classified, both quantitatively and qualitatively, according to cranial index. For quantitative measurement, the percentage of dolichocephalic and mesocephalic indices was similar (40.9% and 40.6% respectively), while, for qualitative measurement, the mesocephalic percentage was higher (50.8%).

**Table 2:** Frequency of cranial categories obtained by qualitative and quantitative measurement in school children between 8–15 years from Envigado, Colombia

	**Direct measurement (quantitative)**		**Indirect measurement (qualitative)**	
	**Frequency**	**Percentage**	**Frequency**	**Percentage**
Dolichocephalic	128	40.9	106	33.9
Mesocephalic	127	40.6	159	50.8
Brachycephalic	58	18.5	48	15.3
Total	313	100	313	100

Upon assessing agreement between direct and indirect measurements of facial index, the Kappa index was 0.189 (95% CI 0.117-0.261), which indicates a poor level of concordance. Among all students assorted as euryprosopic on direct measurement (quantitative), 7 classifications agreed with indirect measurement (qualitative). Within 150 students assorted as mesoprosopic on direct measurement, 57 classifications agreed with the indirect measurement, and from 150 assorted as leptoprosopic, 69 classifications agreed with indirect measurement (Table 3).

**Table 3:** Agreement between the facial index assessed by direct and indirect measurement in school children between 8- 15- year- old from Envigado, Colombia

		**Classification of the facial index based on measurement using and anthropometer (direct)**			**Total children**
		**Euryprosopic**	**Mesoprosopic**	**Leptoprosopic**	
Classification of the facial index based on visual assessment (indirect)	Euryprosopic	7	60	17	84
	Mesoprosopic	5	57	64	126
	Leptoprosopic	1	33	69	103
Total children		13	150	150	313

For the cranial index, a Kappa index of 0.388 (95% CI 0.304-0.473) was obtained, which indicates a poor level. The concordance for facial index as stratified by age varied between 0.004-0.249, and for cranial index ranged between 0.167- 0.541; stratified by gender, female facial index was 0.136 and cranial index: 0.369, and male facial index was 0.032 and cranial index: 0.297; and, stratified by socioeconomic stratum, facial index varied between −0.04- 0.199 and cranial index between 0.25-0.407.Among 128 students classified in the dolichocephalic type based on the direct measurement, 72 were also found to be dolichocephalic by the indirect measurement, 82 out of 127 school boys were found to be mesocephalic in both measurements, and 26 school children were found to be brachycephalic in both types of measurements (Table 4).

**Table 4:** Agreement between cranial index assessed through direct measurement and indirect measurement in school children between 8- 15- year- old from Envigado, Colombia

		**Classification of the cranial index based on measurement using and anthropometer (direct)**			**Total children**
		**Dolichocephalic**	**Mesocephalic**	**Brachycephalic**	
Classification of the cranial index based on visual assessment (indirect)	Dolichocephalic	72	29	5	106
	Mesocephalic	50	82	27	159
	Brachycephalic	6	16	26	48
Total children		128	127	58	313

Regarding the relationship between facial and cranial indices, 21.4% (67/313) of the infants were dolichocephalic and leptoprosopic, 21.1% (66/313) were mesocephalic and leptoprosopic, and 11.5% (36/313) were brachycephalic and mesoprosopic (Table 5).

**Table 5:** Relation between the cranial and facial types, measured using an anthropometer, in school children between 8-15-year- old from Envigado, Colombia

		**Classification of the cranial index based on measurement using an anthropometer (direct)**			**Total children**
		**Dolichocephalic**	**Mesocephalic**	**Brachycephalic**	
Classification of the facial index based on measurement using an anthropometer (direct)	Euryprosopic	3	5	5	13
	Mesoprosopic	58	56	36	150
	Leptoprosopic	67	66	17	150
Total children		128	127	58	313

Kappa’s coefficient to assess intraobserver agreement (initial measurements and measurements performed 5 months later by the same investigator) for cranial index visual measurement was 0.917 ± 0.057, and for facial index 3 was 1.0, indicating an almost perfect match. The intraclass correlation coefficient to assess the intraobserver agreement of the measurement performed with an anthropometer for cranial index was 0.965 (95% CI 0.935-0.982), and for facial index 0.943 (0.894-0.970), indicating an almost perfect agreement.

## Discussion

In this study, the concordance between the direct and indirect measurements for facial and cranial indices was poor, regardless of age, gender and socioeconomic stratum (Kappa index: 0.189 and 0.388, respectively).

Some craniofacial morphology characteristics are associated with certain malocclusions; therefore, they provide the clinician with valuable information for defining a particular treatment plan. The facial type is an instrumental factor for orthodontic treatment, because it can impact the anchorage system, predicts the growth of maxillo-mandibular structures, muscle strength and stability of treatment [13]. During growth process, cranial and facial development can be influenced by a variety of factors, such as: environmental conditions, socioeconomic stratum, race, ethnicity, breathing pattern and nutritional habits [7,14,15]. For example, children from brachycephalic parents show a decreased index when moved to a different country [7]. In addition, in order to establish orthodontic therapy, two basic factors should be considered: 1) assessment of face dimensions: ¿ is the face long or short, leptoprosopic, mesoprosopic or euryprosopic ?, and 2) when performing the intervention, ¿ is a rotational change that may increase or decrease the expression of the dimensions of the face going to be produced?

This study found that the direct classification method for facial index yielded the mesoprosopic and leptoprosopic types as the most predominant, with a percentage of 47.9% each. When compared with other populations, Chileans exhibit a facial index similar to our study [16]; however, the most predominant facial type among Africans is the leptoprosopic [17]. Regarding the cranial type, the greatest percentage of students presented dolichocephalic (40.9%) and mesocephalic (40.6%), compared with other populations. In Africa, the most prevalent cranial type is the dolichocephalic (66.82%) [18],whereas in Southern Iran, the mesocephalic type is the most common (41.98%) [19], and, in India, the brachycephalic type prevails [20]. Comparing studies in growing children, Indian population presents mesocephalic index (77.92%) in males, and brachycephalic index (80.85%) in females [21], while Poland children were brachycephalic (81.45%) [9], as Japanese population [22]. In Iran, 38.6% were euryprosopic and 38% brachycephalic [23].

In the present study, 21.4% of children were found to be dolichocephalic and leptoprosopic, a result which relates to findings reported in the literature, where face anatomy can be determined by the cranial base acting as a framework [13,24]. The growth pattern of cranial and facial indices with growth and structural characteristics of the face exhibits some relationship, which is important to know in order to define an interceptive or corrective orthodontic treatment. The dolichocephalic shape is associated with a leptoprosopic facial type. In contrast, the brachycephalic shape corresponds with an euryprosopic facial type [25]. In dolichocephalic individuals, the brain is relatively narrow and elongated sagittally; this establishes a flatter cranial base, i.e., the angle between the middle and anterior cranial base is wider, which has the following basic implications for the face pattern: 1) the entire naso-maxillary complex is moved forward relative to the jaw due to the rotation of the skull base to the front, and the anterior and middle segments of the cranial base are elongated sagittally; 2) the entire nasomaxillary complex is lower in relation to the mandibular condyle; this causes a downward and backward rotation of the mandible. These people tend to have a retrognathic profile [26,27].

The euryprosopic facial type was the least frequent in this population. Individuals with this type of face generally have strong muscles and morphological features, such as larger transverse size and parallelism between the occlusal and mandibular planes, smaller gonial angle and decreased lower anterior facial height [24]. Those individuals with a brachycephalic type are usually Class III individuals due to a more posterior position of the maxilla, and have a more anterior location of the mandible [28]. However, it is necessary to note that some individuals may present compensations counteracting some malocclusion trends associated with different skeletal types [27-29]. Confirmation of those relationships could be important to determine in a future study.

This illustrates the need to obtain enough data of all individual traits in order to define an adequate therapy plan aiming to reach aesthetic, functional and stability dental-related goals [30].

In this study, a poor level of agreement was found between the direct (anthropometer) and indirect (visual) measurement for both facial and cranial indices. One of the reasons that may explain this finding may be a variation in the location of some landmarks, since it requires palpation representing an underlying skeletal structure; and for the visual assessment, this is made from a superficial soft- tissue, which probably leads to differences in the distances between these points [31]. This may be due to the shape of soft- tissue, which correlates approximately 50% to the form of hard tissue [32], leading to diagnostic mistakes. Another possible explanation is that the percentages used for craniofacial classification were estimated in populations from Europe [12,32], where the Caucasian race is most predominant and exhibits morphological features different from ours (in Colombia, and mainly in Antioquia, where a mixed race prevails). In 2007, [33] Farkas et al. reported significant differences in anthropometric measurements of the craniofacial complex, particularly for the orbit and the nose areas, among white individuals from North America and Afro-Americans between 18–25 -year-old. The authors conclude that there must be separate standards for both groups in order to determine intervention guidelines for the head and face. In clinical practice, decisions hinge on the international anthropometric study of the facial morphology conducted among healthy individuals from Europe (Caucasian), Middle East, Asia and Africa. This study determined face anthropometric measurements (18- 30- year- old). However, it is necessary to establish facial and cranial measurements in our population, in order to be able to define morphological alterations, and to adequately intervene for surgical correction [15]. Additionally, Farkas et al. [5] compared the differences between direct (anthropometrics) and indirect (cephalometries) measurement of dry skulls, and found that the measurements obtained from X-rays were significantly smaller than those obtained from the skull surface. Weinberg et al. [34] compared accuracy between direct anthropometrics and 3D images and did not find significant differences. One of the advantages of the 3D scanning method is that angles, surface areas, volumes and linear distances can be quantified. One of the drawbacks of this method, which is not commonly mentioned, is its high cost.

One of the benefits of direct assessment is the fact that accurate measurements can be obtained without the need to expose patients to ionizing radiation. Also, cost overruns can be avoided, since the only tool needed is an anthropometer. Another advantage observed in the present study was the short time it took to perform direct measurement (about 2 minutes or less to conduct the four measurements necessary for the two indices: facial and cranial), as opposed to other studies which assert that conducting a greater number of measurements in the course of the same exam could become arduous and difficult [35].

An additional strength of this study was the population enrolled: 8 -15- year- old children. In this age range, the most interceptive and corrective treatments are performed [36], since most changes in the craniofacial complex occur during this period. Such changes are first completed in the skull, followed by face width, face depth, and finally, the face length. Due to this facts, they are most likely to be modified [37]. This confirms Farkas assertion, highlighting the importance of developing more detailed anthropometric databases for each ethnical group, due to differences in individuals morphology [15], and for this current case, not only because of racial differences, but also the age group; for example, Russians kids are brachycephalic, while Americans are mesocephalic [9].

According to the literature reviewed, both in Spanish and in English, until it is known, this is the first study that compares a direct measurement method (anthropometer on individuals as opposed to dry skulls) with an indirect method (clinical assessment). Previous studies focused primarily on assessing accuracy between direct and indirect measurement techniques (one of them on dry skulls), which compared photographs, cephalometries or 3D images [31,34,35].

One drawback of this study is that due to its cross-section nature, associations between craniofacial changes taking place during the growth process and the indices could not be established. To do so, a cohort study would be required, in order to determine whether this classification of individuals is modified as their development stage is completed.

## Conclusion

In conclusion, no agreement was found between direct measurement using an anthropometer and indirect measurement via visual assessment. Therefore, the visual classification method (indirect) is not appropriate to assess the cranial and facial indices. Thus, the clinician must use direct measurement in order to obtain reliable data. For future research, the suggestion is to develop a cranial and facial indices for our population, in order to obtain local data about classification ranges.

## Competing interests

The authors declare that they have no competing interests.

## Authors’ contributions

Conception, design of the work, analysis and interpretation of data: AMT, AMQ, JFG, ZVR, NV, PB. Drafting the article: AMT, AMQ, JFG. Revising the paper critically for important intellectual content: AMT, AMQ, JFG, ZVR, NV, PB. Read and approved the final manuscript: AMT, AMQ, JFG, ZVR, NV, PB.

## Pre-publication history

The pre-publication history for this paper can be accessed here:

http://www.biomedcentral.com/1472-6831/14/50/prepub
